# Retrosigmoid Versus Translabyrinthine Approach for Acoustic Neuroma Resection: An Assessment of Complications and Payments in a Longitudinal Administrative Database

**DOI:** 10.7759/cureus.369

**Published:** 2015-10-30

**Authors:** Tyler Cole, Anand Veeravagu, Michael Zhang, Tej Azad, Christian Swinney, Gordon H Li, John K Ratliff, Steven L Giannotta

**Affiliations:** 1 Medical Student, Stanford University School of Medicine; 2 Department of Neurosurgery, Stanford University School of Medicine; 3 Department of Neurological Surgery, Keck School of Medicine of USC

**Keywords:** acoustic neuroma, csf leak, payments, retrosigmoid, translabyrinthine, fat grafting

## Abstract

Object

Retrosigmoid (RS) and translabyrinthine (TL) surgery remain essential treatment approaches for symptomatic or enlarging acoustic neuromas (ANs). We compared nationwide complication rates and payments, independent of tumor characteristics, for these two strategies.

Methods

We identified 346 and 130 patients who underwent RS and TL approaches, respectively, for AN resection in the 2010-2012 MarketScan database, which characterizes primarily privately-insured patients from multiple institutions nationwide.

Results

Although we found no difference in 30-day general neurological or neurosurgical complication rates, in TL procedures there was a decreased risk for postoperative cranial nerve (CN) VII injury (20.2% vs 10.0%, CI 0.23–0.82), dysphagia (10.4% vs 3.1%, CI 0.10–0.78), and dysrhythmia (8.4% vs 2.3%, CI 0.08–0.86). Overall, there was no difference in surgical repair rates of CSF leak; however, intraoperative fat grafting was significantly higher in TL approaches (19.8% vs 60.2%, CI 3.95–9.43). In patients receiving grafts, there was a trend towards a higher repair rate after RS approach, while in those without grafts, there was a trend towards a higher repair rate after TL approach. Median total payments were $16,856 higher after RS approaches ($67,774 vs $50,918, p < 0.0001), without differences in physician or 90-day postoperative payments.

*Conclusions*

Using a nationwide longitudinal database, we observed that the TL, compared to RS, approach for AN resection experienced lower risks of CN VII injury, dysphagia, and dysrhythmia. There was no significant difference in CSF leak repair rates. The payments for RS procedures exceed payments for TL procedures by approximately $17,000. Data from additional years and non-private sources will further clarify these trends.

## Introduction

Surgical intervention remains a viable treatment option for symptomatic or progressively enlarging acoustic neuromas (AN) [1]. The most common surgical approaches to address these tumors are retrosigmoid (RS) and translabyrinthine (TL) [2-3]. A third, middle fossa approach can be considered; however, it has a more limited indication for small intracanalicular tumors [4]. The choice of either the RS or TL approach is dictated by the patient and tumor characteristics, and each approach demonstrates preferential utility in specific situations. While a number of predictive clinical factors have been studied, the relative outcomes of each approach continue to be debated [5-8]. These include tumor size, patient age and overall health status, the anatomy of the vestibule and cerebellopontine angle (CPA), involvement of the brainstem and facial nerve, and the degree of extension into the internal acoustic canal (IAC).

In general, the RS approach is considered more versatile, enabling removal of tumors largely independent of size. In contrast with a TL approach, an RS resection can offer hearing preservation [9]. The RS approach offers the surgeon an improved access to the root entry zone of the acoustic nerve. Disadvantages of the RS approach include the necessity for cerebellar retraction and more limited access to cranial nerve (CN) VII and the cochlear nerves in the distal IAC, increasing the potential for subtotal resection. The TL approach is favored in patients with non-serviceable hearing and in patients with large tumors who have a low probability of hearing preservation [10]. Furthermore, the TL approach offers early identification of the CN VII in the auditory canal during surgery and eliminates any need for cerebellar retraction [11-12].

Although the advantages and disadvantages of different approaches for AN resection have been extensively reported on, a large-scale study to enable their accurate comparison is still greatly needed. The absence of a unified, consistent reporting of variables may contribute to variations in procedure outcomes. For example, in their meta-analysis, Ansari, et al. reported that the RS approach was the most versatile for facial nerve preservation, but it also led to a higher risk of postoperative pain and cerebrospinal fluid (CSF) fistula relative to the middle fossa or TL approach [13]. Meanwhile, Copeland, et al. reported in a recent single-institution, prospective analysis (1999-2012) that the TL approach instead resulted in an increased risk of developing a CSF leak postoperatively [14]. In this retrospective study, we sought to quantify and compare the complete postoperative complication profile of AN resection by retrosigmoid and translabyrinthine approaches with the aid of a nationwide administrative database.

## Materials and methods

### Study design and data source

We performed a retrospective, longitudinal analysis of RS and TL approaches in procedures treating acoustic neuromas from a national database from years 2010–2012. Inpatient and outpatient data were obtained from the Thomson Reuters MarketScan Commercial Claims and Encounters and Medicare Supplemental and Coordination of Benefits databases, administered by Truven Health Analytics. The MarketScan dataset includes data from over 100 payers and includes inpatient, outpatient, and pharmacy services from a range of large employers, health plans, governmental organizations, and public organizations.

### Setting and participants

We identified patients undergoing RS procedures with Common Procedural Terminology (CPT) code 61520 and TL procedures with CPT code 61526. A concurrent International Classification of Diseases (ICD-9) diagnosis code of 225.1 was required to indicate the presence of an acoustic neuroma. Comorbidities were assessed using the Deyo-Charlson and Elixhauser ICD-9 code groupings [15-16]. Supplementary Data 1 contains CPT and ICD-9 codes used in this study and not otherwise defined as part of established comorbidity measures. ICD-9 codes defining complications were only counted if not present within 180 days prior to the index procedure.

We identified surgeons using anonymized payer-specific physician identification numbers within inpatient records from the MarketScan data set. In some cases, a physician may have multiple identification numbers not reconciled among payers; however, due to the anonymization, we considered each physician number as unique. We then calculated the annual volume per individual surgeon identifier.

### Statistical analysis

Tests of significance were performed using two-tailed tests. Categorical variables were analyzed using Fisher’s Exact test; continuous variables were analyzed using Student’s t-test. Due to the examination of a range of complications, significant results (P < 0.05) were adjusted with the Holm-Bonferroni method to determine whether the hypothesis could be rejected. Data preparation and analysis were performed using SAS software (version 9.3; SAS Institute Inc., Cary, NC).

## Results

### Demographics and comorbidities

Within the MarketScan database during the studied period between 2010 and 2012, there were 346 and 130 patients who underwent an RS and TL approach for acoustic neuroma resection, respectively. There was no significant difference between the RS and TL groups for any of the patient factors, comorbidities, or hospitalization characteristics examined, shown in Table 1. This evaluation took into consideration patient age, patient gender, Medicare status, and the presence or absence of the following comorbidities: osteoporosis, tobacco use, congestive heart failure, hypertension, chronic obstructive pulmonary disease, myocardial infarction, diabetes, and obesity.

**Table 1:** Baseline cohort comparison of demographics, hospital-stay characteristics, and medical comorbidities between patients treated by retrosigmoid and translabyrinthine approaches. EPO - exclusive provider organization; HMO - health maintenance organization; POS - point of service; PPO - preferred provider organization; CDHP - consumer-driven health plans; HDHP - hospital-driven health plan

	Retrosigmoid N = 346	Translabyrinthine N = 130	OR (CI)	P-value
Age, mean (SD)	48.8 (12.3)	49.3 (11.9)		0.65
Follow-up, mean, (SD)	315.3 (218.6)	352.7 (224.2)		0.096
Length of stay, mean (SD)	5.4 (5.2)	5.1 (5.7)		0.49
Discharge home, N (%)	304 (86.1)	121 (91)	1.63 (0.84–3.16)	0.17
Demographics and comorbidities, N (%)				
Male	163 (46.2)	58 (43.6)	1.11 (0.74–1.66)	0.68
Medicare	28 (7.9)	8 (6)	0.74 (0.33–1.67)	0.56
Tobacco use	14 (4)	4 (3.1)	0.75 (0.24–2.33)	0.79
Osteoporosis	34 (9.8)	10 (7.7)	0.76 (0.37–1.6)	0.59
Hypertension	118 (34.1)	43 (33.1)	0.96 (0.62–1.46)	0.91
CHF	4 (1.2)	1 (0.8)	0.66 (0.07–5.99)	1.00
COPD	29 (8.4)	8 (6.2)	0.72 (0.32–1.61)	0.56
MI	5 (1.4)	2 (1.5)	1.07 (0.2–5.56)	1.00
Diabetes	35 (10.1)	13 (10)	0.99 (0.5–1.93)	1.00
Obesity	14 (4)	5 (3.8)	0.95 (0.33–2.69)	1.00
Region, N (%)				<.0001
Northeast	115 (32.6)	21 (15.8)		
North Central	78 (22.1)	27 (20.3)		
South	103 (29.2)	74 (55.6)		
West	52 (14.7)	9 (6.8)		
Unknown	5 (1.4)	2 (1.5)		
Insurance plan type, N (%)				0.95
Comprehensive	12 (3.7)	5 (4)		
EPO	6 (1.8)	1 (0.8)		
HMO	35 (10.8)	11 (8.9)		
POS	24 (7.4)	10 (8.1)		
PPO	224 (68.9)	90 (72.6)		
POS with capitation	1 (0.3)	0 (0)		
CDHP	13 (4)	3 (2.4)		
HDHP	10 (3.1)	4 (3.2)		

### Comparative complication and revision procedure rates

There was no significant difference in the incidence of overall general neurological or neurosurgical complications between the two procedures within the 30-day postoperative period, as shown in Table 2. However, the TL procedure was associated with a decreased incidence of specific complications, including postoperative CN VII injury (20.2% vs 10%, p = 0.0096), dysphagia (10.4% vs 3.1%, p = 0.0089), and dysrhythmia (8.4% vs 2.3%, p = 0.022).

**Table 2:** Unadjusted complication rates among patients treated by retrosigmoid and translabyrinthine approaches CN - cranial nerve; NOS - not otherwise specified; (%) percentage reflects proportions within respective RS and TL cohort

	Retrosigmoid N (%)	Translabyrinthine N (%)	OR (CI)	P-value
Wound infection	17 (4.9)	6 (4.6)	0.94 (0.36–2.43)	1.00
Wound dehiscence	6 (1.7)	2 (1.5)	0.89 (0.18–4.44)	1.00
Wound hematoma	11 (3.2)	5 (3.8)	1.22 (0.42–3.58)	0.78
Other wound complication	9 (2.6)	2 (1.5)	0.59 (0.12–2.74)	0.74
Delirium	2 (0.6)	2 (1.5)	2.69 (0.37–19.28)	0.30
Pulmonary embolism	6 (1.7)	0 (0)	-	0.20
Deep venous thrombosis	8 (2.3)	5 (3.8)	1.69 (0.54–5.26)	0.36
Any thromboembolism	4 (1.2)	1 (0.8)	0.66 (0.07–5.99)	1.00
Pulmonary complication	44 (12.7)	18 (13.8)	1.1 (0.61–1.99)	0.76
General neurological complication	74 (21.4)	24 (18.5)	0.83 (0.5–1.39)	0.53
Subarachnoid hemorrhage	8 (2.3)	1 (0.8)	0.33 (0.04–2.64)	0.46
Intracranial hemorrhage NOS	9 (2.6)	2 (1.5)	0.59 (0.12–2.74)	0.74
Precerebral arterial occlusion	2 (0.6)	1 (0.8)	1.33 (0.12–14.83)	1.00
Cerebral artery occlusion	10 (2.9)	3 (2.3)	0.79 (0.22–2.93)	1.00
Transient ischemia attack	3 (0.9)	0 (0)	-	0.57
Acute complication NOS	5 (1.4)	1 (0.8)	0.53 (0.06–4.57)	1.00
Hemiplegia/hemiparalysis	2 (0.6)	1 (0.8)	1.33 (0.12–14.83)	1.00
General neurosurgical complication	50 (14.5)	16 (12.3)	0.83 (0.45–1.52)	0.66
Iatrogenic stroke	6 (1.7)	4 (3.1)	1.8 (0.5–6.48)	0.47
Postop dysrhythmia	29 (8.4)	3 (2.3)	0.26 (0.08–0.86)	0.022
Postop myocardial infarction	9 (2.6)	4 (3.1)	1.19 (0.36–3.93)	0.76
Postop dysphagia	36 (10.4)	4 (3.1)	0.27 (0.1–0.78)	0.0089
Postop CN VII injury	70 (20.2)	13 (10)	0.44 (0.23–0.82)	0.0096
Any complication	184 (53.2)	58 (44.6)	0.71 (0.47–1.06)	0.10
30-days all-cause readmission	73 (21.1)	21 (16.2)	0.72 (0.42–1.23)	0.25

While there was no difference in the rate of lumbar drain placement (11.2% vs 6.9%, p = 0.23) or surgical repair of CSF leak (11.2% vs 11.5%, p = 0.87), the use of a fat graft during surgical repair of a CSF leak was significantly higher in the TL approach (19.8% vs 60.2%, p < 0.0001), shown in Table 3.

**Table 3:** 30-day readmission rate and adjunctive procedures, including tissue grafting, drain placement, and CSF leak repair and SRS between retrosigmoid and translabyrinthine approaches CSF - cerebral spinal fluid; AN - acoustic neuroma; SRS - stereotactic radiosurgery (* Limited to procedures recorded within the MarketScan database, not indicative of absolute AN surgeon volume)

	Retrosigmoid N (%)	Translabyrinthine N (%)	OR (CI)	P-value
Intraoperative tissue grafting	70 (19.8)	80 (60.2)	6.1 (3.95–9.43)	< 0.0001
Lumbar drain placement	39 (11.2)	9 (6.9)	0.59 (0.28–1.26)	0.23
Within 30 days	33 (9.5)	9 (6.9)	0.71 (0.33–1.53)	0.47
With graft	5 (7.2)	6 (7.7)	1.07 (0.31–3.66)	1.00
Without graft	34 (12.1)	3 (5.8)	0.44 (0.13–1.5)	0.23
Repair of CSF leak	39 (11.2)	15 (11.5)	1.04 (0.55–1.95)	0.87
Within 30 days	31 (8.9)	14 (10.8)	1.24 (0.64–2.41)	0.60
With fat graft	11 (15.9)	5 (6.4)	0.36 (0.12–1.1)	0.11
Without fat graft	28 (10)	10 (19.2)	2.14 (0.97–4.73)	0.093
Surgeons performing ≥ 2 AN procedures annually*	25 (7.2)	13 (10)	1.44 (0.71–2.91)	0.34
Postoperative SRS	16 (4.7)	7 (5.5)	1.17 (0.47–2.92)	0.81

In patients with grafts, the need for a CSF leak repair trended towards being higher in the RS group (15.9% vs 6.4%, p = 0.11). However, in those without fat grafts, it was higher in the TL group (10% vs 19.2%, p = 0.093). Of note, we also found that patients who underwent the TL approach and received fat grafting were less likely to develop CSF leak requiring surgical intervention (6.4% vs 19.2%, p = .047). In contrast, there was a trend towards increased risk of CSF leak requiring surgical intervention with fat grafting in patients who received an RS approach (15.9% vs 10%, p = 0.20).

### Procedure payments, length of stay and readmission rates

The RS approach resulted in higher median total payments ($67,774 vs $50,918, p = 0.0004) and hospital payments ($50,351 vs $36,855, p = 0.0025). There was no significant difference between the two procedures for median physician payments ($8,575 vs $7,499, p = 0.17) or aggregate 90-day postoperative payments ($18,607 vs $12,513, p = 0.15).  There was no difference in the length of hospital stay, follow-up, or discharge home. These findings are shown in Table 4.

**Table 4:** Comparative median immediate and 90-day post-discharge payments between retrosigmoid and translabyrinthine approaches IQR - interquartile range

	Retrosigmoid N = 346	Translabyrinthine N = 130	P-value
Hospital payments, median (IQR)	50351 (46702)	36855 (34438)	0.0025
Physician payments, median (IQR)	8575 (8326)	7499 (5670)	0.17
Total payments, median (IQR)	67774 (50374)	50918 (35572)	0.0004
90-day post-discharge payments, median (IQR)	18607 (39829)	12513 (39939)	0.15

### Surgical volume and adverse events

Surgeons who are recorded in the MarketScan database as performing at least two AN procedures annually had a decreased incidence of CN VII injury (18.7% vs 2.6%, p = .0072) and postoperative dysphagia (8.9% vs 2.6%, p = .24), as well as a trend towards decreased need for repair of CSF leak (12% vs 2.6%, p = 0.11), compared to surgeons performing fewer than two procedures per year. These results are shown in Table 5. Though not statistically significant, we also noted a strong trend for surgeons who performed more than two procedures per year to utilize intraoperative fat grafting (44.7% vs 29.5%, p = .066). The annual volume of procedures that a given surgeon performed did not impact the incidence of postoperative dysrhythmia.

**Table 5:** Pertinent complication rates and adjunctive procedures, stratified by annual surgeon experience with any acoustic neuroma resection approach CN - cranial nerve; CSF - cerebral spinal fluid. (* Limited to procedures recorded within the MarketScan database, not indicative of absolute AN surgeon volume)

	Surgeons Performing < 2 Procedures Annually*	Surgeons Performing ≥ 2 Procedures Annually*	OR (95% CI)	P-value
N treated patients	438	38		
Postop CN VII injury	82 (18.7)	1 (2.6)	0.12 (0.02–0.87)	0.0072
Postop dysphagia	39 (8.9)	1 (2.6)	0.28 (0.04–2.07)	0.24
Postop dysrhythmia	30 (6.8)	2 (5.3)	0.76 (0.17–3.29)	1.00
Intraoperative tissue grafting	130 (29.5)	17 (44.7)	1.94 (0.99–3.79)	0.066
Repair of CSF leak	53 (12)	1 (2.6)	0.2 (0.03–1.47)	0.11

## Discussion

This national sample of patients undergoing surgical intervention for acoustic neuromas between 2010 and 2012 compared the complications and costs between these two common approaches. We observed that an RS approach, relative to a TL one, was associated with an increased rate of postoperative facial nerve injury (18.7% vs 2.6%) and postoperative dysphagia (8.9% vs 2.6%). Although the frequency of CSF leak was equivalent between approaches, the choice of intraoperative fat grafting reflected each procedure’s respective needs for adequate dural repair [17]. Total hospital payments for a TL approach were significantly lower than that required for an RS approach ($36,855 vs $50,351) and did not markedly impact payments associated with postoperative care.

Follow-up analyses did not indicate that variations in the length of stay or lumbar drain placement contributed to the difference in costs between RS and TL approaches. However, the diverging postoperative complication rates, particularly in relationship to provider volume, may play a role. Prior work has reported that high volume centers achieve a lower average cost of hospitalization [18-19]. Low volume surgeons, using the more versatile retrosigmoid, may encounter a higher complication rate, potentially leading to the increased use of hospital resources not available in the MarketScan database [20]. Viewed collectively, our findings can inform practitioners by determining the composite risks of AN surgery in the broader neurosurgical community, as opposed to evaluating results from just one or a few centers. Otherwise, the general neurological complication rates between each surgical approach were comparable.

The rate of facial nerve dysfunction associated with AN surgery is an important clinical outcome. Affecting one out of five RS patients in our analysis, it was the most prominent individual complication in the early postoperative phase. The TL approach is traditionally thought to provide a more anatomically accommodating view of the facial nerve and, therefore, can limit facial nerve injury [21]. Existing studies have reported comparable facial nerve dysfunction rates [22-24]. In a series of 200 operations utilizing the RS approach, Samii, et al. reported a 19% rate of facial nerve dysfunction (House-Brackmann Grade IV and V) with a mean follow-up of 24 months [25].

Meanwhile, other studies emphasizing additional parameters or with longer follow-up have reported varying complication rates. For example, Ansari, et al. reported that at 23.3 months median follow-up, there was no difference between surgical approaches when treating smaller tumors and that an RS approach can actually have better facial nerve preservation rates when treating larger tumors [13]. Since facial nerve dysfunction is known to potentially recover within the first postoperative year, complication rates between RS and TL may become statistically masked over time [26-29]. By limiting our study to the early postoperative phase, we avoid any confounding temporal variations in reporting.

Although less common than facial nerve dysfunction, postoperative injury to the neighboring vagus nerve, presenting as dysphagia, is another important clinical outcome for patients and providers. The complication is again largely limited to the early postoperative phase, with follow-up surveys indicating that 71% recover from their vagal palsies. Those with palsies were no more likely than those who never presented with palsies to have long-term difficulties related to their voice or swallowing [30]. Nevertheless, patients continue to express concern over facial and vagal nerve injuries and continue to perceive cranial nerve preservation as an indicator of surgical success [21, 31]. Fortunately, these often transient postoperative symptoms do not ultimately impact patients' quality of life outcomes [32]. Perhaps, also related to the involvement of the vagus nerve, we identified a higher rate of dysrhythmia in RS procedures; however, this did not affect the rate of postoperative transient ischemic attacks, arterial occlusion, or myocardial infarction.

Interestingly, the rate of CSF leak is comparable between surgical approaches. Although there remains no consensus on the best graft protocol for preventing postoperative CSF leaks, there is encouraging data showing that fat grafting can reduce the rate of a dural leak to 0-7.4% [33-37]. Within our data, the choice to include a graft did vary based on the surgical approach. There was an increased trend of incorporating intraoperative fat grafts for TL approaches, though this was markedly lower (60.2%) than we expected, given the relative consensus within the neurosurgical literature around fat grafting with employment of the TL approach [20]. The lower than expected rate of fat grafting in TL procedures may be a result of varying institutional coding practices. Nevertheless, the importance of grafting in TL approach was reaffirmed when we found that use of a graft was protective against the risk of a dural leak requiring surgical intervention in TL approaches. Given this finding and the existing neurosurgical literature, we encourage this additional closure technique when possible.

Finally, we observed significant variation in inpatient financial impact with AN resections. The difference in median total payment exceeded $15,000 between the two approaches, and this margin is likely to play an increasingly larger role in clinical decision-making as the cost of AN surgery continues to rise [38-39]. Since recurrence is already very low, future changes in the financial and clinical cost of surgery will likely heavily reflect the increasing costs of the primary surgery [38]. These costs of RS surgery also can be weighed alongside the well-characterized risks of hearing loss, a known absolute complication, in TL surgery. Ansari, et al. indicated that the probability of hearing loss is about 64.3% in tumors < 1.5 cm and 71.6% in those between 1.5 and 3.0 cm when resected by the RS approach [13]. Thus, when microsurgery is indicated and tumor features cannot guide the surgical approach, early postoperative risk of facial nerve dysfunction, vagal nerve dysfunction, and increased hospital payments of RS approach should collectively be considered.

Our study has several limitations, which should be noted. The MarketScan dataset does not characterize tumor size or location (e.g.. intracanalicular involvement), which frequently guides the surgical approach. Our analysis is intended as a broad overview of the comparative outcomes of the procedures independent of preoperative evaluation and planning. Secondly, the MarketScan database is limited in assessing surgical volume or providers, as it only captures procedures whose payment is provided by insurers within the database. Our assessment of volume is, therefore, more reflective of relative, and not absolute, volume differences. We are also not able to differentiate between academic and private practices, which frequently vary in case distribution and complexity. Outcome differences may be attributable to surgeons' experience with acoustic neuroma management and the approaches for resection. In our analysis of CSF leaks, it is important to note we consistently discussed the rates of CSF leak requiring surgical intervention. We are unable to report the objective rates, as the ICD-9 codes that may provide that information is unreliable. Furthermore, because cranial nerve palsies have been shown to resolve and hospital costs are most prominent during the initial hospitalizations, we believe our data appropriately focuses on the symptomatically and resource-intensive period relevant for accurate quality assessment.

Our report, which is accompanied by the limitations of all retrospective studies, provides an updated, large-scale survey of current AN surgical care. These results can serve to inform future evaluations of both community- and academic-based practices.

## Conclusions

From a nationwide, administrative database spanning 2010-2012, AN resection by TL approach was found to have a lower rate of CN VII injury, dysphagia, and dysrhythmia compared to that by RS approach. Although there was no significant difference in CSF leak repair rates, the rate for tissue grafting differed by approach. Surgeons performing multiple AN resections had lower rates of CN VII dysfunction and lower CSF leak repair rates, independent of the procedure. The mean payment for RS procedures was significantly greater than that for TL procedures; however, there was no difference in post-hospitalization costs.

## Appendices

Supplementary Data 1. CPT and ICD-9-CM coding utilized for cohort selection, comorbidity assessment (not elsewhere defined as part of established comorbidity measures), and post-operative outcome analysis; MI, myocardial infarction; CN, cranial nerve; CSF, cerebrospinal fluid

Comorbidity or Postoperative Outcome Coding

Tobacco use (ICD-9) - 305.1, V15.82, 989.84, 649.0

Osteoporosis (ICD-9) - 733, V17.81, 731.3, V82.81

General neurological complication (ICD-9) - 430, 431, 432, 433, 434, 435, 436, 438.2, 438.3, 438.4, 438.5

General neurosurgical complication (ICD-9) - 997

Dysrhythmia (ICD-9) - 427, 426.1, 426, 426.3, 426.4, 426.5, 426.6, 426.7, 426.8

MI (ICD-9) - 410, 412, 998.0, 997.1, 411, 429.7

Dysphagia (ICD-9) - 787.2

CN VII injury (ICD-9) - 351

Lumbar drain placement (CPT) - 62272

Tissue grafting (CPT) - 20926

Repair of CSF leak (CPT) - 61618, 61619, 62100, 69670

Stereotactic radiosurgery (CPT) - 61796, 61797, 61798, 61799, 61800, 63620, 63621
